# Early recognition and response to increases in surgical site infections using optimized statistical process control charts—the Early 2RIS Trial: a multicenter cluster randomized controlled trial with stepped wedge design

**DOI:** 10.1186/s13063-020-04802-4

**Published:** 2020-10-28

**Authors:** Deverick J. Anderson, Iulian Ilieş, Katherine Foy, Nicole Nehls, James C. Benneyan, Yuliya Lokhnygina, Arthur W. Baker

**Affiliations:** 1 Duke Center for Antimicrobial Stewardship and Infection Prevention, Durham, NC USA; 2 Healthcare Systems Engineering Institute, Northeastern University, Boston, MA USA; 3 Department of Biostatistics, Duke University School of Medicine, Durham, NC USA

**Keywords:** Surgical site infection, Surveillance, Statistical process control, Feedback, Outbreak detection

## Abstract

**Background:**

Surgical site infections (SSIs) cause significant patient suffering. Surveillance and feedback of SSI rates is an evidence-based strategy to reduce SSIs, but traditional surveillance methods are slow and prone to bias. The objective of this cluster randomized controlled trial (RCT) is to determine if using optimized statistical process control (SPC) charts for SSI surveillance and feedback lead to a reduction in SSI rates compared to traditional surveillance.

**Methods:**

The Early 2RIS Trial is a prospective, multicenter cluster RCT using a stepped wedge design. The trial will be performed in 29 hospitals in the Duke Infection Control Outreach Network (DICON) and 105 clusters over 4 years, from March 2016 through February 2020; year one represents a baseline period; thereafter, 8–9 clusters will be randomized to intervention every 3 months over a 3-year period using a stepped wedge randomization design. All patients who undergo one of 13 targeted procedures at study hospitals will be included in the analysis; these procedures will be included in one of six clusters: cardiac, orthopedic, gastrointestinal, OB-GYN, vascular, and spinal. All clusters will undergo traditional surveillance for SSIs; once randomized to intervention, clusters will also undergo surveillance and feedback using optimized SPC charts. Feedback on surveillance data will be provided to all clusters, regardless of allocation or type of surveillance. The primary endpoint is the difference in rates of SSI between the SPC intervention compared to traditional surveillance and feedback alone.

**Discussion:**

The traditional approach for SSI surveillance and feedback has several major deficiencies because SSIs are rare events. First, traditional statistical methods require aggregation of measurements over time, which delays analysis until enough data accumulate. Second, traditional statistical tests and resulting *p* values are difficult to interpret. Third, analyses based on average SSI rates during predefined time periods have limited ability to rapidly identify important, real-time trends. Thus, standard analytic methods that compare average SSI rates between arbitrarily designated time intervals may not identify an important SSI rate increase on time unless the “signal” is very strong. Therefore, novel strategies for early identification and investigation of SSI rate increases are needed to decrease SSI rates. While SPC charts are used throughout industry and healthcare to improve and optimize processes, including other types of healthcare-associated infections, they have not been evaluated as a tool for SSI surveillance and feedback in a randomized trial.

**Trial registration:**

ClinicalTrials.govNCT03075813, Registered March 9, 2017.

## Administrative information

Note: the numbers in curly brackets in this protocol refer to SPIRIT checklist item numbers. The order of the items has been modified to group similar items (see http://www.equator-network.org/reporting-guidelines/spirit-2727-statement-defining-standard-protocol-items-for-clinical-trials/__;!!OToaGQ!_EXmkNIa7FXJ6Jgpz6jAg0-5b4y1YtykPoGvMbNiq-XcNuK6n1xNKyptp7XW0y93TTg95kA$).

**Table:** 

Title {1}	Early recognition and response to increases in surgical site infections using optimized statistical process control charts—The Early 2RIS Trial: a multicenter cluster randomized controlled trial with stepped wedge design
Trial registration {2a and 2b}.	ClinicalTrials.gov - NCT03075813 All registry items are included in the body of the protocol.
Protocol version {3}	2.1
Funding {4}	United States Federal Government - Agency for Healthcare Quality and Research (AHRQ) R01 HS23821; National Institutes of Health (NIH) 5T32AI100851–02
Author details {5a}	Anderson, Foy, Baker – Duke Center for Antimicrobial Stewardship and Infection Prevention, Division of Infectious Diseases, Duke University School of Medicine, Durham, NC Lokhnygina – Department of Biostatistics and Bioinformatics, Duke University School of Medicine Ilieş, Nehls, Benneyan – Healthcare Systems Engineering Institute, Northeastern University, Boston, MA
Name and contact information for the trial sponsor {5b}	AHRQ
Role of sponsor {5c}	No role outside of financial support

## Introduction

### Background and rationale {6a}

Surgical site infections (SSIs) are the most common and costly healthcare-associated infections (HAIs) in the USA [1–4]. More than 150,000 patients acquire a SSI each year and suffer from adverse outcomes, including longer hospitalizations and increased mortality than patients without SSIs [5–7]. In total, SSIs cost the US healthcare system more than $3 billion annually [4, 8]. Over the past decade, hospitals across the USA have spent considerable time and resources optimizing SSI prevention processes. While most hospitals have greatly improved compliance with important process measures, improved process performance has not led to decreased rates of SSI [9, 10]. As a result, innovative strategies to prevent SSIs are greatly needed.

Statistical process control (SPC) is an analytic approach that combines time series analysis methods with graphical presentation of data to determine whether a process or rate exhibits “common cause” natural variation or “special cause” variation due to circumstances that have not previously been inherent in the process [11]. In other words, SPC methods help separate “noise” from a true signal. Commonly employed in manufacturing and other industries, SPC methods have emerged as useful tools for identifying and analyzing changes in HAI rates [12–15]. To date, however, SPC methods are not commonly utilized in a rigorous manner to provide real-time surveillance of HAIs such as SSIs.

Feedback of SSI data to surgical personnel is a cornerstone of SSI prevention [16] and is well proven to lead to lower rates of SSI [17–20]. Indeed, the feedback of surveillance data to surgeons has repeatedly been shown to improve surgical patient outcomes, including SSI [17–20]. However, current surveillance and feedback strategies are significantly limited. SPC surveillance and feedback of SSI data can overcome these limitations and decrease rates of SSI. SPC surveillance is designed to rapidly identify important increases in the rate and occurrence of SSIs. When a signal is identified, an investigation can be performed to provide rapid review and feedback to determine if process changes are required, potentially reducing the risk of SSI for subsequent patients.

## Objectives {7}

To measure the effectiveness of surveillance using optimized SPC methods and feedback on rates of SSI compared to traditional infection surveillance methods and feedback.

### Primary objective

1. To determine if hospital clusters randomized to SSI surveillance with optimized SPC methods and feedback collectively have lower rates of SSI compared to hospital clusters randomized to traditional SSI surveillance methods and feedback.

### Secondary objectives

1. To determine if hospital clusters randomized to SSI surveillance with optimized SPC methods and feedback collectively have lower rates of superficial-incisional, deep-incisional, organ/space, and/or complex SSI compared to hospital clusters randomized to traditional SSI surveillance methods and feedback.
2. To determine and compare the number of signals identified using optimized SPC methods and traditional surveillance methods over the 3-year post-baseline period.
  - Descriptive – overall numbers; then summarize per cluster per month
  - For comparison – develop standardized rate, likely number of signals per 100 procedures performed
3. To estimate and compare the proportion of signals that led to investigations using optimized SPC methods versus traditional surveillance methods, including the number of true positive signals, false positive signals, positive predictive value, sensitivity, and specificity of each method.
4. To summarize the preventability score for SSIs reviewed during the study and compare between treatment arms.
5. To compare the timing of true positive signal identification between the two surveillance strategies.
  - Average time between signals
  - Proportion of true positive signals in which optimized SPC methods found the signal first.

## Trial design {8}

The Early 2RIS Trial is a prospective, multicenter cluster randomized controlled trial using stepped wedge design. Baseline data will be collected for 12 months. The active component of the trial will be performed in 29 hospitals in the Duke Infection Control Outreach Network (DICON) hospitals and 105 clusters over 3 years, from March 2017 through February 2020.

## Methods: participants, interventions, and outcomes

### Study setting {9}

All study hospitals participate in DICON (http://dicon.medicine.duke.edu/). DICON is a network of over 60 community hospitals in North Carolina, South Carolina, Georgia, Virginia, Florida, and West Virginia that provides access to consultative services from infection prevention experts, data analyses and benchmarking, and educational materials designed by faculty at the Duke University School of Medicine. Each site has a contract (Infection Prevention Program Development Services Agreement) with Duke, which includes a data use agreement (DUA) and business agreement (BAA). Routine network activities, including regular visits by DICON liaison nurses, data reports, and education, will continue throughout the study. All participating hospitals submitted letters of support for inclusion in the study.

### Eligibility criteria {10}

#### Inclusion criteria

All patients who undergo one of 13 targeted procedures at 29 study hospitals will be included in the analysis. These procedures were selected because they are frequently performed in community hospitals and/or are associated with particularly adverse outcomes if complicated by SSI. Eligible procedures will be categorized by type at each hospital using ICD10 codes published by the US CDC’s National Healthcare Safety Network (NHSN) [21]. Clusters will be constructed at each participating hospital based on the type of surgical procedure performed (Table 1). Clusters were constructed to ensure that surgeons who perform similar types of procedures were grouped together to limit potential bias. *These clusters will be the units for randomization and analysis*.

**Table 1:** Procedures included in each cluster

Cluster	Procedure
Cardiac	Coronary artery bypass graft
	Cardiac valve replacement
GI	Colon
	Herniorrhaphy
Joint	Knee arthroplasty
	Hip arthroplasty
OB-GYN	Cesarean section
	Hysterectomy
	Vaginal hysterectomy
Spine	Spinal fusion
	Laminectomy
Vascular	Carotid endarterectomy
	Peripheral venous bypass

#### Exclusion criteria

DICON hospitals that did not submit a letter of support for participating in the study will be excluded. Patients not undergoing one of the 13 procedure types at the 29 study hospitals will be excluded from the analysis. Patients undergoing one of the 13 procedure types at the 29 study hospitals with infection present at the time of surgery will be excluded from the analysis.

### Who will take informed consent? {26a}

Patients will not be consented as part of this study. A waiver of informed consent was granted by the Duke University Health System IRB.

### Additional consent provisions for collection and use of participant data and biological specimens {26b}

N/A. Patients will not be consented for this trial.

## Interventions

### Explanation for the choice of comparators {6b}

**Fig. 1:**
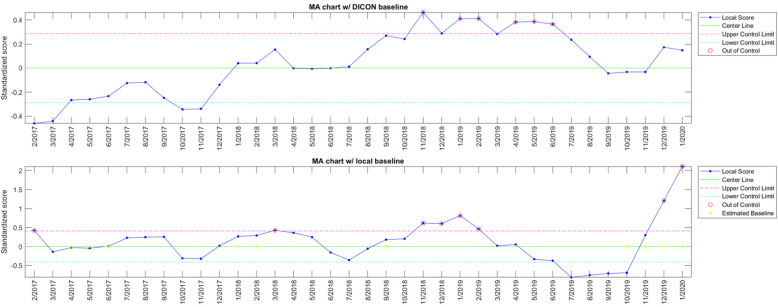
Example SPC charts used for SSI surveillance in the Early 2 RIS Trial; Chart A (top) is a moving average (MA) chart using DICON baseline rates and Chart B (bottom) is a moving average (MA) chart using local hospital rates

Optimized SPC charts were identified following systematic evaluation of 13 years of data from 58 DICON hospitals (1.35 million procedures and 14,147 SSIs) [22]. Optimized SPC methods include a logical disjunction of 2 different variations of moving average (MA) charts (i.e., signals are generated whenever either chart features an out-of-control point). Chart A evaluates local SSI rates against medium-term historical SSI rates (18-month reference period with a 6-month lag) for the same procedure type across all other DICON hospitals and aggregates these differences using a 12-month MA. Chart B evaluates current SSI rates against recent SSI rates (3-month baseline interval with a 3-month lag) for the same procedure type at the same hospital and aggregates these differences using a 6-month MA (Fig. 1). Therefore, while the former chart can detect long-term differences between local and DICON SSI rates, the latter is highly sensitive in identifying sustained, short-term local increases in SSI incidence, irrespective of the network-wide DICON rate. Both charts employ a narrow control limit of 1 standard deviation and are thereby tuned for screening potential signals, rather than detecting only definite signals and rejecting those unlikely to represent definitive outbreaks. Correspondingly, this specific chart combination reached sensitivities of 0.9 and 0.88, and specificities of 0.67 and 0.75, on the training (initial 12 years) and validation (last year of data) subsets, respectively. In contrast, traditional SSI surveillance has a reported sensitivity of only 33% to 65% [23, 24].

Importantly, SPC surveillance should be viewed as an adjunct to traditional surveillance. That is, traditional surveillance will still be performed in both arms to identify and document SSIs as they occur in study hospitals. In the intervention arm, optimized SPC surveillance will be applied to SSI surveillance data after traditional surveillance is performed to rapidly identify if the data entered and new SSI rates are not in line with expected rates (a “signal”).

### Intervention description {11a}

Data entered locally by infection preventionists at DICON hospitals are transmitted to the DICON Surgical Database per routine practices. Data submitted to the DICON Surgical Database will undergo scheduled weekly analysis by optimized SPC methods. If a signal is generated, study personnel in DICON will be notified to adjudicate the signal and determine if further action is required.

### Criteria for discontinuing or modifying allocated interventions {11b}

N/A. Criteria for discontinuing or modifying allocated interventions were not developed.

### Strategies to improve adherence to interventions {11c}

N/A. Subjects and surgeons will be blinded to study arm.

### Relevant concomitant care permitted or prohibited during the trial {11d}

N/A. Individual patient care will not be impacted by this trial.

### Provisions for post-trial care {30}

N/A. Care will be provided by primary teams throughout the study, not research personnel.

### Outcomes {12}

Primary endpoint (domain: healthcare-associated infection):

- Specific measurement – SSI rate, calculated as the number of SSIs per 100 procedures
- Specific metric – Differences in the rates of SSI between the intervention compared to traditional surveillance and feedback alone
  - SSIs will be defined using standard NHSN definitions
    i. DICON personnel train local infection preventionists about how to use and interpret SSI definitions. Thus, standard definitions and methods will be used at all study hospitals
  - Cluster-level risk adjustment will be performed using median surgical volume [25] and median NHSN Risk Index (an operation- and patient-specific risk score that predicts SSI) [26, 27] per cluster
- Method of aggregation – Mean rates will be summarized for each cluster for each 3-month study “step”

Secondary endpoints:

- Several secondary outcomes will be compared between clusters receiving intervention and clusters receiving traditional surveillance and feedback
  - Stratified analysis of primary outcome – SSI rates stratified by type of SSI (superficial-incisional, deep-incisional, or organ/space).
  - Descriptive outcomes
    ◦ Description of and difference in number and type of signals
    ◦ Difference in number of investigations of increased rates of SSI
    ◦ Total number and differences in proportion of signals that led to investigations
    ◦ Proportion of SSIs determined to be potentially preventable, based on preventability score
    ◦ Timing of signals

### Participant timeline {13}

All hospitals and clusters will participate for the entire duration of the study, including 1 year of baseline data collection and 3 years of implementation of intervention through a stepped wedge approach. Patients that undergo surgical procedures of interest during the 4-year study period will be followed for up to 90 days post-operatively, per CDC SSI definitions and standard methods.

**Fig. 2:**
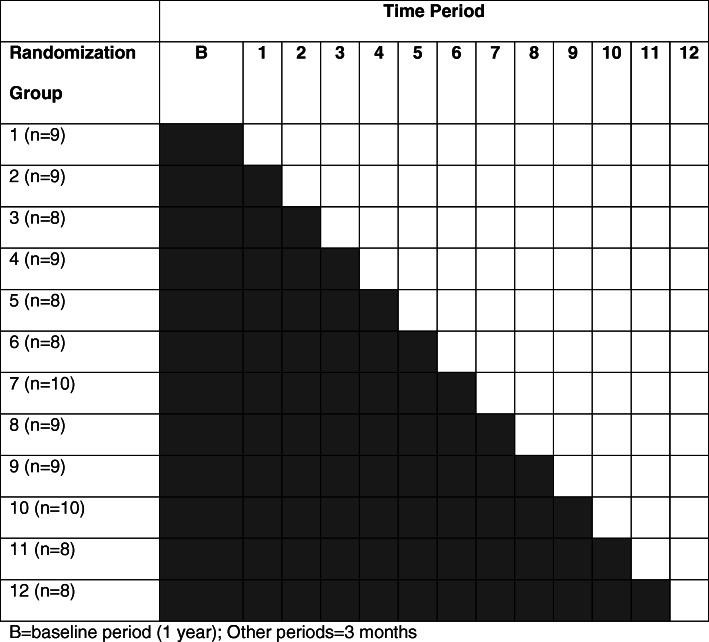
Schematic for stepped wedge design for the Early Recognition and Response to Increases in Surgical Site Infection (Early 2RIS) Trial. Gray = control, during which hospitals will receive traditional SSI surveillance, including biannual data reports. Any signals identified in biannual reports or detected by local personnel will undergo further investigation. White = intervention, during which hospital clusters will receive feedback from traditional surveillance and signals generated by applying optimized SPC methods to SSI surveillance data

Randomization will occur at the cluster level within hospitals. Clusters within each hospital will potentially be changed to intervention during 12 “steps” (Fig. 2).

### Sample size {14}

In our power calculation, we utilized 3 years of pilot data from 101 clusters of procedures in 29 DICON hospitals (including 1622 SSIs following 154,554 procedures). Power was evaluated via a simulation study where for each cluster, log (SSI rate) was generated from a multivariate normal distribution with the following assumptions: (1) cluster-specific SSI rate for traditional surveillance phases calculated from the pilot data (average rate including procedures from all clusters was 1.33%), (2) residual variance for log (SSI rate) of 0.76, (3) within-cluster correlation of 0.36, and (4) between cluster correlation of 0.39 in the same time step and 0.2 in different steps. Based on these assumptions, a study with 101 clusters in 29 DICON hospitals, 12 steps, and an average of 127 procedures per cluster per 3-month step would have 90% power to detect a 25% decrease in the SSI rate between optimized SPC methods and traditional surveillance.

### Recruitment {15}

No specific recruitment will be completed for this study. All participating hospitals are part of DICON.

## Assignment of interventions: allocation

### Sequence generation {16a}

Randomization and allocation sequence will be performed using computer-generated random numbers. However, at most, one cluster per hospital will be changed to the intervention during any individual step; all remaining clusters will continue to receive standard, traditional surveillance and feedback. Once randomized to intervention, clusters will receive surveillance from both optimized SPC and standard methods for the remainder of the study. At the last step, the remaining cluster within each hospital (if any) will begin the intervention.

### Concealment mechanism {16b}

N/A. No concealment was performed.

### Implementation {16c}

The study statistician (Lokhnygina) will develop the allocation sequence. The study coordinator (Foy) will assign clusters to interventions as per the randomization scheme.

## Assignment of interventions: blinding

### Who will be blinded {17a}

SSI signals prospectively identified using optimized SPC methods during the intervention period will undergo blinded review to ensure that signal adjudication occurs without knowledge of which hospital cluster generated the signal. Following review, the study coordinator will unblind the signal, and, if the hospital cluster is randomized to intervention, the study team will proceed with the actions required to appropriately respond to the identified increase in SSIs. If the hospital cluster is a control cluster, then the study coordinator will document the signal, but no further action will be taken.

### Procedure for unblinding if needed {17b}

N/A. No unblinding was performed.

## Data collection and management

### Plans for assessment and collection of outcomes {18a}

Routine data on surgical procedures and SSIs will be collected via a standardized limited dataset, per routine DICON practices (Table 2) [28]. No identifiable patient or surgeon data are transmitted to the DICON Surgical Database.

**Table 2:** Variables in the DICON Surgical database

All surgical patients	Hospital
	Type of procedure
	Patient identifier
	Date of procedure
	Age
	Sex
	Surgeon identifier
	Start/stop times
	ASA score
	Wound class
	Risk index
	SSI (yes/no)
Patients with Infection	Date of infection
	Type of SSI
	Location at diagnosis
	Organism

When a signal is identified, data will be collected on the rationale for signal adjudication (action vs. no action). If a signal requires action, additional data will be collected for further investigation, per routine protocol.

The majority of data collection will occur through methods already developed and utilized by study hospitals. In brief, each hospital routinely submits limited datasets to the DICON Surgical Surveillance Database, including all variables listed in Table 2. Data definitions and data collection methods are standardized across DICON hospitals. Following signal adjudication, additional data will be collected in a REDCap database to document actions and rationale.

### Plans to promote participant retention and complete follow-up {18b}

N/A. Follow-up will be mandatory and completed through routine SSI surveillance methods.

### Data management {19}

DICON IT personnel will be responsible for data management required for the DICON Surgical Database, per routine DICON practices. The study coordinator will be responsible for documentation required for the study.

REDCap is a toolset and workflow methodology for electronic collection and management of research and clinical trial data. Both REDCap and REDCap Survey systems provide secure, web-based applications that can be used for a variety of types of research, provide an intuitive interface for users to enter data, and have real-time validation rules (with automated data type and range checks) at the time of data entry. These systems offer easy data manipulation and an automated export mechanism to common statistical packages (e.g., SPSS, SAS, Stata, R/S-Plus).

The REDCap program will serve as the portal for data entry by the study coordinator. Data entered into this database will be password protected and only accessible by study personnel. All access to this secure separate database will be monitored and logged.

Surgical data, including SSI data, will be maintained in the DICON Surgical Surveillance Database. Data related to signal adjudication and response will be entered into REDCap databases.

### Confidentiality {27}

This study does not involve the collection of PHI.

### Plans for collection, laboratory evaluation, and storage of biological specimens for genetic or molecular analysis in this trial/future use {33}

N/A. No specimens will be collected for this trial.

## Statistical methods

### Statistical methods for primary and secondary outcomes {20a}

Data will be summarized using standard statistical methods. Analyses will be performed using the intention-to-treat strategy.

#### Primary analyses

The primary outcome will be analyzed using a generalized estimating equations approach with a Poisson or negative binomial model (if overdispersion is detected using Poisson), which will model SSI rate at a cluster level as a function of time (step) and intervention phase (traditional vs. optimized SPC surveillance), while accounting for within-cluster correlation over time and between-cluster correlation within each study hospital. This model will utilize the data from all steps, including the baseline period. To account for any potential residual confounding, we will consider including cluster-level risk-adjustment variables in the model, as described in the previous section. Inference about the model parameter corresponding to the intervention phase will be used to address the main hypothesis (2.1). In case the number of zeroes per cluster exceeds the number of zeroes modeled by Poisson (or negative binomial) distribution, we will consider a zero-inflated Poisson (negative binomial) model.

#### Secondary analyses

The outcome of superficial-incisional, deep-incisional, organ/space, and/or complex SSIs will be analyzed similarly to the primary outcome. The rate of signals per 100 procedures performed will be compared between optimized SPC and traditional surveillance methods using a similar approach as for the primary outcome. Sensitivity and positive predictive value to identify important increases in SSI rates (defined as signals that lead to investigations) will be compared between optimized SPC and traditional surveillance methods using chi-square or Fisher’s exact test, as appropriate. For a subset of true positive signals that are generated by both methods, average time between signals will be summarized, and a proportion of true positive signals in which optimized SPC methods identified the signal first will be estimated. The remaining secondary outcomes will be analyzed using summary statistics only.

Full details of the statistical analysis will be specified in the statistical analysis plan prior to the study database lock.

### Interim analyses {21b}

No interim analyses are planned for this trial.

### Methods for additional analyses (e.g., subgroup analyses) {20b}

No further subgroup analyses are planned other than described above.

### Methods in analysis to handle protocol non-adherence and any statistical methods to handle missing data {20c}

As all study data are collected as part of routine practices, missing data are anticipated to be minimal. Though not anticipated, patients with missing SSI data will be excluded.

### Plans to give access to the full protocol, participant level-data, and statistical code {31c}

The full study protocol will be available via request, through our website, and through the journal that ultimately publishes the trial result. Summary data will be available by request. Participant level data is restricted by BAAs in place with participating hospitals and will not be made available.

## Oversight and monitoring

### Composition of the coordinating center and trial steering committee {5d}

N/A. No coordinating center or steering committee will be developed for the trial.

### Composition of the data monitoring committee, its role and reporting structure {21a}

N/A. No data monitoring committee will be created.

### Adverse event reporting and harms {22}

N/A. No reporting structure will be developed or required.

### Frequency and plans for auditing trial conduct {23}

N/A. No auditing will be performed for this trial.

### Plans for communicating important protocol amendments to relevant parties (e.g., trial participants, ethical committees) {25}

IRB renewal occurs annually. This and all other interactions with the DUHS IRB will occur through routine mechanisms.

## Dissemination plans {31a}

We plan to disseminate the results of this trial at national and international meetings that target important audiences: infectious diseases physicians, surgeons, epidemiologists, infection preventionists, healthcare executives, and policymakers. To target the infectious diseases physicians, surgeons, and epidemiologists, we plan to present the results at IDWeek, a large, annual, international infectious diseases conference. To target policymakers, we will present at annual AHRQ meetings and will discuss our results with colleagues at the US Centers for Disease Control (CDC). Results will be prepared and edited into manuscript(s) for peer-review publication. Relative to knowledge translation, we will advise stakeholders (e.g., the AHRQ, CDC, and CDC’s Division of Healthcare Quality Promotion (DHQP)) of results for dissemination.

## Discussion

Surgical site infections (SSIs) cause significant patient suffering and are the most common and costly healthcare-associated infections (HAIs) in the USA [1–7]. Innovative strategies to prevent SSI are needed.

Feedback of SSI data to surgical personnel is a cornerstone of SSI prevention [16] and is well proven to lead to lower rates of SSI [17–20]. Indeed, the feedback of surveillance data to surgeons has repeatedly been shown to improve surgical patient outcomes, including SSI [17–20]. Traditional SSI surveillance at individual hospitals involves a multi-step process: data collection, rate calculation (typically on a quarterly or semiannual basis), and feedback. Rates can be compared to previous rates at the hospital and/or to external benchmarks, such as those established by the NHSN [29] or programs like the National Surgical Quality Improvement Project (NSQIP).

The traditional approach for SSI surveillance and feedback has several major deficiencies because SSI is a low-frequency event. First, the traditional approach is slow. Traditional statistical methods require aggregation of measurements over time, which delays analysis until enough data accumulate [30]. In practice, hospital epidemiologists are often *told* about a problem (e.g., from a perceptive surgeon) rather than discovering the problem via ongoing “real-time” data analysis. Second, traditional statistical tests and resulting *p* values are difficult to interpret, due to sporadic outcomes and small numbers. Thus, standard analytic methods that compare average SSI rates between arbitrarily designated time intervals may not identify an important SSI rate increase unless the “signal” is very strong. Third, analyses based on average SSI rates during predefined time periods have limited ability to rapidly identify important, real-time trends. For example, a cluster of SSIs may occur during 1 month, but this “signal” could be diluted by accrual of additional data in subsequent months prior to the next scheduled analysis. Finally, the use of external benchmarks such as NHSN or NSQIP is challenging because of cost, delayed reports, use of historical data, lack of feedback of actionable items, and concentration on a few, specific procedures.

*Statistical process control (SPC) methods specifically address and overcome all of these deficiencies.* More specifically, SPC methods can be semi-automated to run more regularly, are more easily interpretable due to graphical display, and can be specifically designed to identify important changes in the setting of infrequent outcomes. To date, however, SPC methods are not commonly utilized in a rigorous manner to provide real-time surveillance of HAIs such as SSIs. Furthermore, SPC strategies have not been evaluated as strategy to prevent SSI using a randomized controlled trial.

## Trial status

**Table:** 

Version	Date	Notes
1.0	March 1, 2017	Original approved protocol
2.0	May 17, 2017	Updated objectives, hypotheses, statistical section
2.1	April 17, 2018	Eliminated typo from objectives; corrected Schematic/Description of Study Design

Recruitment began on March 1, 2017, and will end on February 29, 2020. Data collection will continue for 90 days thereafter as per routine surveillance methods. We planned to submit this protocol manuscript in 2019 before the end of recruitment, but many competing priorities led to delays in submission. Of note, no changes to the protocol occurred following the update in 2018 described above.

## Abbreviations

DICON: Duke Infection Control Outreach Network
HAI: Healthcare-associated infection
MA: Moving average
NHSN: National Healthcare Safety Network
NSQIP: National Surgical Quality Improvement Program
SPC: Statistical process control
SSI: Surgical site infection
CDC: US Centers for Disease Control and Prevention
AHRQ: US Agency for Healthcare Quality and Research
